# Association of *pth* Variants with Elevated Persister Fractions in Clinical *Escherichia coli* Isolates

**DOI:** 10.3390/pathogens15080875

**Published:** 2026-08-20

**Authors:** Lei Xu, Jinxi Yue, Bingxue Ning, Kangxinhe Zhang, Guoyang He, Manqi Xiao, Zixuan Zhang, Xinyi Zheng, Xiaodan Yan, Hebin Liao

**Affiliations:** 1 School of Laboratory Medicine & Translational Medicine Research Center, North Sichuan Medical College, Nanchong 637000, China; 2 Department of Clinical Laboratory, Affiliated Hospital of North Sichuan Medical College, Nanchong 637000, China; 3 Institute of Infection and Immunity, Henan Academy of Innovations in Medical Science, Zhengzhou 450000, China

**Keywords:** *pth*, persister, antibiotic tolerance, clinical *Escherichia coli*

## Abstract

Persister cells pose severe threats and serve as the primary drivers of chronic and recurrent infections. Nevertheless, clinical laboratories rarely quantify persister fractions of pathogenic bacteria due to the limited number of detection approaches and scarce available detection biomarkers. In our prior study, we identified a transposon insertion mutant of the essential gene *pth*. The growth curve, lag time and persister fraction measurements indicated that the *pth*-Tn strain exhibited slow growth with a markedly elevated persister cell proportion. Transcriptomic and metabolomic analyses revealed that the *pth*-Tn strain exhibited typical persister phenotypic characteristics, including reduced energy metabolism, membrane biogenesis, substrate transport and translational activity, among others. The *pth* mutations in host-transmitted *E. coli* could serve as indicators of persister levels, as also demonstrated in vitro. Thus, we identified seven point mutations in *pth* among clinical isolates. Compared with clinical isolates harboring the wild-type *pth* gene, the G109S mutants displayed higher persister fractions. Multiple linear regression models revealed that the G109S mutation was independently associated with elevated persister levels. Collectively, these findings reveal an association between the *pth* G109S mutation and elevated persister levels in clinical isolates.

## 1. Introduction

The Global Burden of Disease Study 2019 released by Antimicrobial Resistance Collaborators indicated that bacterial infections have become the second leading cause of death worldwide [1]. The latest research report published by the same consortium projected that by 2050, 8.22 million global deaths would be linked to antimicrobial resistance (AMR), among which 1.91 million deaths would be directly attributable to AMR [2]. Persister cells provide a protective niche and opportunity for bacteria to undergo further evolutionary variation and develop drug resistance [3]. Therefore, investigations on persister cells are highly important for addressing antimicrobial resistance. Persister cells are a heterogeneous phenotypic subpopulation within bacterial communities. They can survive exposure to lethal concentrations of antibiotics and resume proliferation once antibiotic pressure is eliminated [4]. Clinically, persister cells constitute one of the primary drivers of chronic and recurrent infections, including recurrent urinary cystitis and nephritis, tuberculosis, cystic fibrosis-associated chronic pulmonary infections, and medical device-related biofilm infections [5,6,7,8].

The formation mechanisms of persister cells are complex and include but are not limited to toxin–antitoxin (TA) systems, (p)ppGpp, c-di-GMP, the SOS response, and various stress conditions [9,10,11,12,13,14]. Persister cells constitute only a minor subset of the total bacterial population, regardless of the underlying formation mechanism. Owing to the absence of a unified pathway governing persister formation and the extremely low abundance of persister cells, specific biomarkers and detection techniques for persister identification are severely lacking [4,15,16]. Moreover, most studies on persister cells rely on laboratory model strains, whereas the actual characteristics of clinical isolates remain insufficiently investigated [17,18,19,20]. Therefore, clinical laboratories rarely conduct persister assays on clinical bacterial isolates and thus cannot provide clinicians with valuable references regarding persister levels.

**Figure 1:**
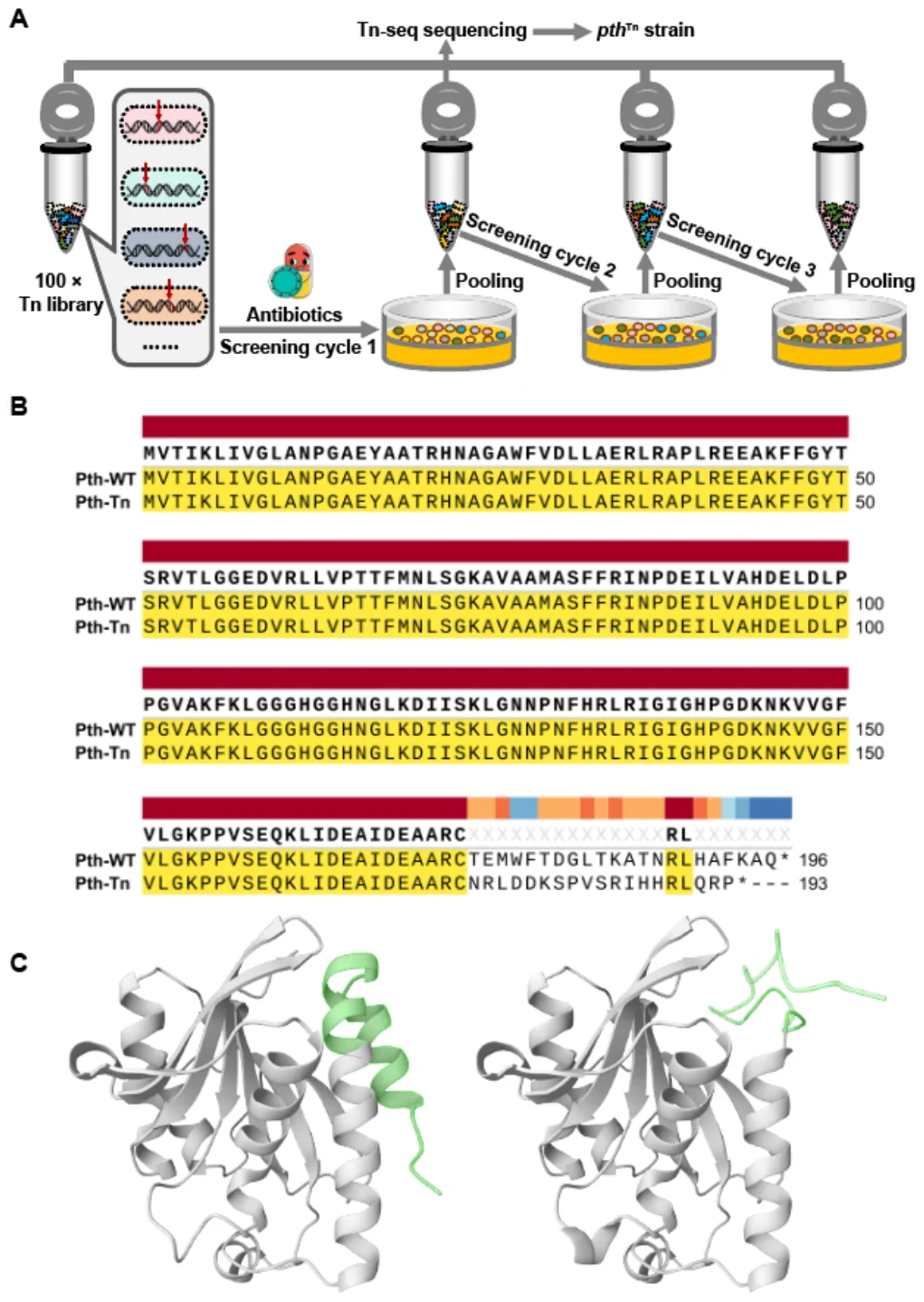
Identification of the *pth*-Tn mutant via transposon library screening. (**A**) Schematic of the isolation of the *pth*-Tn mutant via multiround antibiotic screening from the MG1655 transposon mutant library. (**B**) Protein sequence alignment of Pth-Tn and Pth-WT. (**C**) Predicted protein spatial structures of Pth-Tn and Pth-WT, with structural modeling performed via AlphaFold.

In a previous study conducted by our team, we performed multiple rounds of antibiotic-based persister screening using the MG1655 transposon mutant library (Figure 1A). We found that the persister fraction of mutants with mutations in the essential gene *pth* was markedly elevated. Peptidyl-tRNA hydrolase (Pth) hydrolyzes ribosome-free peptidyl-tRNAs that drop off the ribosome during protein synthesis or as a result of ribosome stalling and contain one or more amino acids [21,22,23]. Pth can also catalyze the release of premature peptidyl moieties from peptidyl-tRNA molecules trapped in stalled 50S ribosomal subunits. In this way, Pth maintains sufficient free tRNA and 50S ribosomes inside cells [24]. Pth is indispensable for normal bacterial growth. Certain point mutations in the *pth* gene reduce its enzymatic activity or substrate-binding capacity and inhibit cellular growth [23,25,26,27]. Therefore, the elevated persister fractions observed in these *pth* mutant strains are biologically reasonable. These observations are consistent with those of a previous study in which the persister levels of the *pth* mutants G101D and A170P were increased approximately 100-fold relative to that of the parental strain [28].

Artificial screening under laboratory conditions can generate loss-of-function *pth* mutants [28]. Accordingly, we hypothesize that naturally occurring *pth* mutations also arise in human pathogenic bacteria circulating in patients, increasing the persister fractions of clinical isolates. To verify this hypothesis, we analyzed 122 clinically isolated pathogenic *Escherichia coli* strains and identified seven distinct clinical point mutations in the *pth* gene. Among these clinical isolates, strains carrying *pth* G109S substitutions displayed significantly higher persister fractions than did wild-type *pth* isolates.

## 2. Materials and Methods

### 2.1. Bacterial Strains and Growth Conditions

All the strains and plasmids used in this study are described in Table S1. Unless otherwise specified, the strains were cultured in Luria broth (LB) medium under routine growth conditions at 37 °C and 200 rpm. When necessary, the bacteria were cultured with chloramphenicol at a final concentration of 25 μg/mL or gentamicin at 20 μg/mL.

Clinical isolates of *Escherichia coli* were collected from the Department of Clinical Laboratory, Affiliated Hospital of North Sichuan Medical College, and all the strains were identified as *Escherichia coli* by a VITEK MS Fully Automated Microbial Mass Spectrometry Identification System (bioMérieux, Marcy-l’Étoile, France). The minimum inhibitory concentration (MIC) values of these strains were determined using a VITEK 2 XL automated microbial identification and susceptibility testing system (bioMérieux, Marcy-l’Étoile, France). This research followed the guidelines of the Medical Ethics Committee of the Affiliated Hospital of North Sichuan Medical College (2026ER382-1).

### 2.2. Strain Construction

To construct the *pth*-Tn strain, the generation of the target DNA mutant strain involved the use of λ-Red-mediated gene replacement [29]. In brief, three fragments, including the stop codon of the *pth*-Tn gene and its 500 bp upstream sequence amplified from the *pth*^Tn^ strain, *cat* (chloramphenicol resistance cassette), and a 500 bp downstream sequence of the *pth* coding region amplified from the MG1655Δ*araD-B* strain, were fused into a single DNA fragment. This fragment was subsequently electroporated into MG1655Δ*araD-B* carrying the pSIM6 plasmid. Cells carrying the pSIM6 plasmid were subjected to 20 min of heat shock at 37 °C before being used to generate competent cells. Transformants were screened on LB plates supplemented with 20 μg/mL chloramphenicol. Afterward, the transformants were verified by PCR and DNA sequencing to confirm the presence of the *pth*-Tn mutation. Positive verified strains were cultured at 37 °C to remove the pSIM6 plasmid. The overnight bacterial culture was supplemented with 20% glycerol and stored at −80 °C.

To construct *pth* point mutant strains, a codon-optimized sequence of the *pth* gene was chemically synthesized by Sangon Biotech (Shanghai, China) as a template (Text S2). Four fragments, including the 500 bp upstream sequence of the target mutation site amplified from MG1655Δ*araD-B*, the fragment between the target mutation site and the stop codon amplified from the codon-optimized *pth* gene, *cat* (chloramphenicol resistance cassette), and the 500 bp downstream sequence of the *pth* coding region amplified from strain MG1655Δ*araD-B*, were fused into a single DNA fragment. This fragment was used to construct the *pth* point mutant strains as described above.

For plasmid construction, gene cloning was performed using seamless cloning technology. Briefly, the backbone of the pBAD vector, in which the original ampicillin resistance cassette was replaced with a gentamicin resistance gene, was amplified as one fragment. The full-length *pth* gene sequence, including a 278 bp upstream fragment of the start codon, was amplified from the genomic DNA of strain MG1655 as the second fragment. The two amplified fragments were subjected to in vitro recombination using the 2× MultiF Seamless Assembly Mix (Cat. No. RK21020, ABclonal Technology, Wuhan, China). The recombination product was transformed into competent *E. coli* DH5α cells. Transformants were screened on agar plates containing 20 μg/mL gentamicin, and successful gene cloning was verified by Sanger DNA sequencing.

### 2.3. Persister Counting Assay

The test strains were inoculated into LB medium and cultured at 37 °C with shaking at 220 rpm until they reached the target growth state. Before the antibiotic treatment, the test cultures were serially diluted with PBS, and an adequate amount of bacterial suspension was spotted on LB plates to count the number of colony-forming units (CFUs). For the antibiotic treatment, the test bacterial cultures were diluted 1:20 into LB medium supplemented with the corresponding antibiotics and incubated at 37 °C and 220 rpm for 3 h. The 3 h treatment reaches the second phase of biphasic bacterial killing, which enables reliable measurement of persister cell abundance (Figure S1). The antibiotics used were 150 μg/mL ampicillin (Amp), 150 μg/mL ceftazidime (Caz), 150 μg/mL cefoperazone (Cps), 15 μg/mL netilmicin (Net), 20 μg/mL tobramycin (Tob), 3 μg/mL ofloxacin (Ofx), 6 μg/mL ciprofloxacin (Cip) and 2.5 μg/mL meropenem (MEM). The bacterial suspensions were then centrifuged at 5000× *g* for 2 min at room temperature to collect cell pellets, which were subsequently resuspended in sterile PBS. After serial dilution, appropriate dilutions were plated on LB agar for CFU counting. The persister fraction was calculated as the ratio of the number of CFUs recovered after respective antibiotic treatments to the initial number of CFUs before antibiotic exposure. At least three biological replicates were performed for each experiment.

### 2.4. Growth Curve Assay

Strains were inoculated into LB medium and cultured overnight at 37 °C with shaking at 220 rpm. The overnight culture was diluted 1:100 into fresh liquid LB medium and incubated at 37 °C and 220 rpm. Optical density (OD) values at 600 nm were measured every 1.5 h. At least three biological replicates were performed for each experiment.

### 2.5. Lag-Time Assay

The lag-time assay was performed as previously described [30]. Briefly, the strains were inoculated into LB medium and cultured overnight at 37 °C with shaking at 220 rpm. The overnight culture was diluted 1:100 into fresh liquid LB medium and incubated at 37 °C and 220 rpm for 3–5 h to the mid-exponential phase. Appropriate bacterial suspensions were serially diluted and spread onto LB agar plates, with approximately 100–200 colonies per plate. The plates were placed on an Epson Perfection V33 scanner and then incubated at 37 °C. The scanner was controlled using ScanningManager software [30], with scanning performed every 15 min. The plate images were analyzed with ImageJ (version 1.54f) to determine the time point at which colonies appeared. At least three biological replicates were performed for each experiment.

### 2.6. Quantitative Real-Time PCR Assay

The MG1655 Δ*araD-B* WT and *pth* point mutant strains were inoculated into LB medium and cultured overnight at 37 °C with shaking at 220 rpm. The overnight cultures were diluted 1:100 into fresh liquid LB medium and incubated at 37 °C and 220 rpm for 12 h until the stationary phase. Bacterial pellets were harvested, washed with PBS, and immediately processed for total RNA extraction using the Bacteria RNA Extraction Kit (Cat. No. R403, Vazyme, Nanjing, China). Total RNA was used for cDNA synthesis using ABScript Neo RT Master Mix for qPCR with gDNA Remover (Cat. No. RK20433, Abclonal). qPCR reactions were performed with 2× Universal SYBR Green Fast qPCR Mix (Cat. No. RK21203, Abclonal). Primers for amplification of the 16S rRNA were as follows: 5′-TAGAATTCCAGGTGTAGCGG-3′ and 5′-GGGTATCTAATCCTGTTTGCTC-3′. Primers for amplification of the *pth* gene were as follows: 5′-GATTAAATTGATTGTCGGCCTG-3′ and 5′-TGACTCGCGAAGTATAACCA-3′. The relative mRNA levels of *pth* were quantified using the 2^−ΔΔCt^ method, with 16S rRNA serving as the internal reference.

### 2.7. Bulk RNA-seq and Untargeted Microbial Metabolomics Assays

The MG1655 Δ*araD-B* WT and *pth*-Tn::Cm strains were inoculated into LB medium and cultured overnight at 37 °C with shaking at 220 rpm. The overnight cultures were diluted 1:100 into fresh liquid LB medium and incubated at 37 °C and 220 rpm. MG1655 Δ*araD-B* WT reached mid-exponential phase at approximately 3 h, whereas *pth*-Tn::Cm required about 5 h, with an OD_600_ of around 0.8 for mid-exponential-phase cultures. Bacterial pellets were harvested, washed with PBS, and immediately frozen for subsequent analysis. Three biological replicates were prepared per group for transcriptomic profiling. Four biological replicates were included for each group in metabolomic analysis. For the metabolomic assay, three quality control (QC) samples were prepared by pooling extracts randomly selected from a subset of individual test samples.

#### 2.7.1. Untargeted Microbial Metabolomics Assay

Frozen samples preserved at −80 °C were thawed gradually in an ice bath and subsequently homogenized using a tissue grinder at a frequency of 30 Hz for 20 s. In brief, 40 mg of homogenized sample was mixed with 400 μL of methanol-aqueous solution (4:1, *v*/*v*) containing internal standards, followed by 3 min of vigorous vortex oscillation. The mixture was subjected to three repeated freeze-thaw cycles: each cycle consisted of 5 min of freezing in liquid nitrogen, 5 min of cooling on dry ice, ice-bath thawing, and 2 min of vortex mixing. After the pretreatment procedure was complete, the sample solution was centrifuged at 12,000 rpm and 4 °C for 10 min. A total of 300 μL of the supernatant was collected, incubated at −20 °C for 30 min, and then recentrifuged under the same temperature and rotational speed conditions for 3 min. Ultimately, 200 μL of the clarified supernatant was transferred for subsequent liquid chromatography-mass spectrometry (LC-MS) detection.

All the samples were detected in both positive and negative electrospray ionization modes with a unified elution gradient program. Chromatographic separation was carried out on a Waters ACQUITY Premier HSS T3 column (1.8 μm particle size, 2.1 mm × 100 mm; Waters Corporation, Milford, MA, USA). The mobile phase consisted of solvent A (0.1% formic acid aqueous solution) and solvent B (0.1% formic acid acetonitrile solution). The gradient elution procedure was as follows: 2 min, 5–20% solvent B; 3 min, 20–60% solvent B; 1 min, 60–99% solvent B (maintained for 1.5 min); rapid reversion to 5% solvent B within 0.1 min; and a 2.4 min equilibration stage. The column oven temperature was stabilized at 40 °C, the mobile phase flow rate was set to 0.4 mL/min, and the sample injection volume was 3 μL for each test.

Raw mass spectral data were converted to mzML standard format via ProteoWizard software (version 3.0.7414). The XCMS software package (version 4.10.1) was used for peak extraction, spectral alignment and retention time calibration. Metabolic features with true-absence-of-peak-signal rates exceeding 50% across all experimental groups were eliminated to ensure data reliability. For the retained features, a hybrid imputation strategy was applied for data complementation: for features with zero-intensity values in more than 50% of experimental samples, missing signals were supplemented with one-fifth of the minimum spectral intensity; for features with zero-intensity values in less than 50% of experimental samples, the k-nearest-neighbor (KNN) algorithm was utilized for data imputation. Support vector regression (SVR) was employed to calibrate and correct the peak intensity values of metabolic features. Qualitative annotation of screened and normalized metabolic features was performed by matching the acquired MS/MS fragment spectra with the in-house spectral database of MetWare Biotechnology Co., Ltd. (Wuhan, China), together with integrated public spectral databases (e.g., Metlin, HMDB, KEGG, MoNA, and MassBank) and an AI-predicted library. Metabolites were categorized according to metabolite annotation confidence levels (1a, 1b, 2, 3, 4). Briefly, Level 1a: Metabolite identification based on high-confidence matching against authentic standards with MS1, RT and MS2 parameters. Level 1b: Metabolite identification based on moderate-confidence matching against authentic standards with MS1, RT and MS2 parameters. Level 2: Metabolite identification based on high-confidence matching of MS1, RT and MS2 parameters (without authentic standards). Level 3: Metabolite identification based on moderate-confidence matching of MS1, RT and MS2 parameters (without authentic standards). Level 4: Metabolite identification based on matching of MS1 and RT parameters only (features at this level were excluded from differential analysis). Matches from the AI-predicted library were only used to support Level 2–4 annotations.

#### 2.7.2. RNA-Sequencing

Strand-specific transcriptome libraries were constructed in strict accordance with a previously reported protocol [31]. In detail, total bacterial RNA was isolated using the phenol-chloroform extraction method, and the RNA integrity and total yield were precisely assessed and quantified with an Agilent 2100 Bioanalyzer system. For prokaryotic library preparation, ribosomal RNA (rRNA) was first removed from the total RNA sample, and the purified RNA was further recovered through ethanol precipitation. The processed RNA was then subjected to fragmentation, and first-strand complementary DNA (cDNA) was synthesized using random hexamer primers. In the process of second-strand cDNA synthesis, dUTP was adopted to replace dTTP in the reaction system to ensure strand specificity. A series of follow-up experimental procedures, including terminal end repair, 3′-end A-tailing, sequencing adapter ligation, target fragment size screening, polymerase chain reaction (PCR) amplification and product purification, were sequentially performed to obtain qualified strand-specific sequencing libraries. Finally, all the constructed libraries were subjected to paired-end 150 (PE150) sequencing on the Illumina X Plus platform.

Raw sequencing reads were preprocessed with fastp software (version 0.23.4) to remove low-quality sequences. Specifically, reads containing adapter contaminants, poly-N ambiguous bases, and low-quality bases were filtered out, yielding high-quality clean reads. Based on the refined clean data, the Q20/Q30 quality scores and GC content distribution were statistically evaluated for data quality validation. All downstream bioinformatics analyses were performed using the qualified clean datasets. The processed clean reads were then aligned to the species reference genome using Bowtie2 (version 2.5.2), allowing a maximum of two mismatches per read; the other parameters were set to default values. The read abundance corresponding to each annotated gene was calculated with the featureCounts tool. Raw integer read counts generated by featureCounts were used as input for differential expression analysis using the DESeq2 R package. Gene expression levels were further standardized into fragments per kilobase of transcript per million mapped reads (FPKM) values by correcting for gene length and sequencing depth. FPKM values were used solely for data visualization and descriptive presentation, and were not applied for statistical testing of differential expression across sample groups. The clusterProfiler package was subsequently employed to conduct Gene Ontology (GO) functional annotation and Kyoto Encyclopedia of Genes and Genomes (KEGG) pathway enrichment analysis for the identified differentially expressed genes.

### 2.8. Multiple Linear Regression

Multiple linear regression analysis was performed to identify independent genetic factors associated with bacterial persister fractions. The outcome variable was the persister fraction obtained after ceftazidime or meropenem exposure. The primary predictor was *pth* G109S mutant. Covariates including *pth* N144D mutant, susceptibility to multiple antibiotics and strain source information, were adjusted in the model. For missing data handling, isolates with missing values in any variable were excluded from the regression analysis. After data filtering, the final sample size was *n* = 85 for the ceftazidime model and *n* = 103 for the meropenem model. All statistical analyses were conducted using Microsoft Excel software. Residual outputs were generated for visual inspection of model assumptions. Formal quantitative model-diagnostic tests such as multicollinearity assessment were not performed.

### 2.9. Statistical Analysis

Statistical analysis was performed in GraphPad Prism 9 software for Windows. Significance was ascertained by two-tailed Student’s *t* test or the Mann-Whitney U test. The error bars represent the standard deviations of the mean from at least three biological replicates. A value of *p* < 0.05 was considered significant. “*” indicates significant differences (*, *p* < 0.05; **, *p* < 0.01; ***, *p* < 0.001; ****, *p* < 0.0001).

## 3. Results

### 3.1. A Mutant Strain of the Essential Gene pth Was Identified via Library Screening

To screen genes involved in persister formation, we previously performed a transposon library screening study (unpublished data). Briefly, we utilized a transposon mutant library of the *Escherichia coli* K-12 strain MG1655 to collect persister cells under antibiotic treatment. The harvested persister population was subjected to the next round of persister enrichment, and a total of three rounds of screening were performed (Figure 1A). After Tn-seq analysis of the screened transposon mutant library, we unexpectedly identified an insertion mutant of the essential gene *pth*, designated *pth*^Tn^ (Figure 1A). Sequence analysis indicated that transposon insertion modified the C-terminal 22 amino acids of the essential *pth* gene in this mutant strain (Figure 1B). Pth is a peptidyl-tRNA hydrolase that hydrolyzes ribosome-free peptidyl-tRNAs and catalyzes the release of premature peptidyl moieties from peptidyl-tRNA molecules. We were prompted to investigate structural changes caused by the *pth*-Tn mutation in the Pth enzyme. The protein tertiary structure was modeled using AlphaFold. We found that the C-terminal α-helix secondary structure of the mutant Pth was converted into an intrinsically disordered region (Figure 1C). The C-terminal α-helix of Pth serves as the core structural element responsible for Pth recognition of the tRNA TΨC stem [27]. Residues H188, N185 and K182 within this helix enable the enzyme to anchor into the minor groove of the tRNA TΨC stem. Consequently, mutations in the C-terminal α-helix may severely impair the substrate-binding capacity of Pth.

### 3.2. The pth-Tn Mutation Promotes Persister Formation

**Figure 2:**
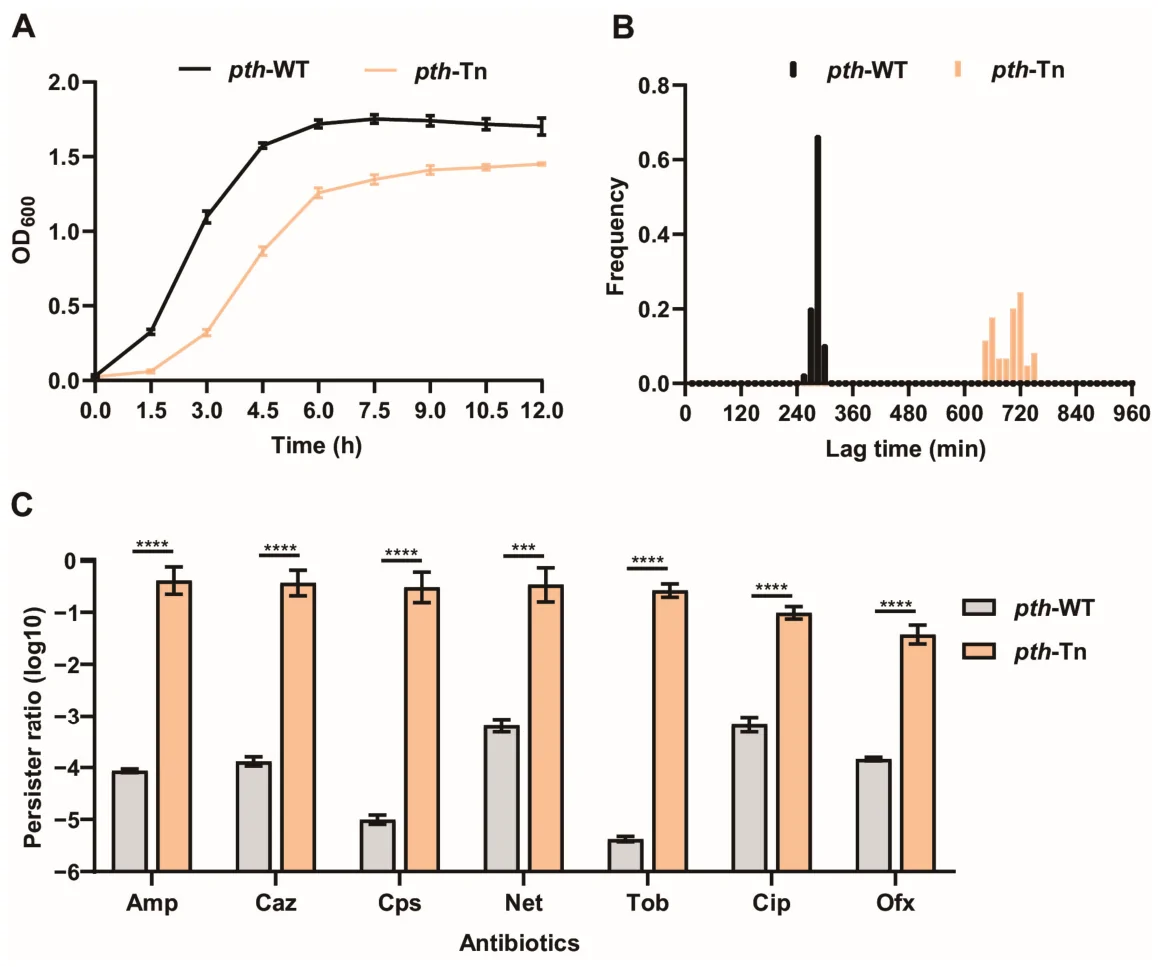
The *pth*-Tn mutant exhibited elevated persister cell levels. (**A**) Growth curves of the *pth*-Tn and *pth*-WT strains. Overnight cultures were diluted 1:100 in fresh LB medium and incubated at 37 °C with shaking at 220 rpm. The optical density at 600 nm (OD_600_) was measured every 1.5 h (*n* = 3). (**B**) Lag times of *pth*-Tn and *pth*-WT. Overnight cultures were diluted 1:100 in fresh LB medium and incubated at 37 °C with shaking at 220 rpm until the mid-exponential phase. The bacterial suspensions were serially diluted appropriately, spread onto LB agar plates, and subjected to lag time measurement (*n* = 3). (**C**) Persister cell assays of the *pth*-Tn and *pth*-WT strains. Overnight cultures were diluted 1:100 into fresh LB medium and incubated at 37 °C with shaking at 220 rpm until they reached the stationary phase, after which persister cells were detected. Bacteria were treated with antibiotics for 3 h at the following concentrations: 150 μg/mL ampicillin (Amp), 150 μg/mL ceftazidime (Caz), 150 μg/mL cefoperazone (Cps), 15 μg/mL netilmicin (Net), 20 μg/mL tobramycin (Tob), 3 μg/mL ofloxacin (Ofx), and 6 μg/mL ciprofloxacin (Cip). Significance was ascertained by two-tailed Student’s *t* test. The error bars represent the standard deviations of the mean from at least three biological replicates. A value of *p* < 0.05 was considered significant. “*” indicates significant differences ***, *p* < 0.001; ****, *p* < 0.0001).

To further explore how the *pth*-Tn mutant with a truncated C-terminal α-helix influences bacterial physiology, we generated a corresponding mutant in the MG1655 Δ*araD-B* background at the initial stage of this study. Overnight cultures of the parental and *pth*-Tn mutant strains were subcultured at a 100-fold dilution into fresh LB medium for growth curve analysis. The results revealed that the parental strain took approximately 2 h to reach the mid-logarithmic phase, whereas the *pth*-Tn mutant strain required approximately 4 h to reach the mid-logarithmic phase (Figure 2A). The growth of the *pth*-Tn mutant was markedly delayed. Lag time directly reflects the dormancy and growth status of bacterial populations [30,32,33]. Therefore, we further measured the lag time when subculturing mid-exponential-phase cultures of the parental strain and the *pth*-Tn mutant. The results revealed that the lag time of the parental strain ranged from 255–300 min, whereas the lag time of the *pth*-Tn mutant ranged from 645–750 min, with a significant difference between the two strains (Figure 2B).

The growth restriction and prolonged lag time suggest that the proportion of persister cells may increase significantly. A persister counting assay demonstrated that following Amp killing, the persister ratio of the parental strain was 10^−4.06±0.04^, whereas that of the *pth*-Tn mutant reached 10^−0.39±0.26^, corresponding to a 4726-fold increase (*p* < 0.0001). For Caz killing, the persister ratio of the parental strain was 10^−3.88±0.09^, whereas that of the *pth*-Tn mutant reached 10^−0.43±0.25^, corresponding to a 2805-fold increase (*p* < 0.0001). For Cps killing, the persister ratio of the parental strain was 10^−5.00±0.09^, whereas that of the *pth*-Tn mutant reached 10^−0.52±0.29^, corresponding to a 30,000-fold increase (*p* < 0.0001). For Net killing, the persister ratio of the parental strain was 10^−3.19±0.12^, whereas that of the *pth*-Tn mutant reached 10^−0.47±0.32^, corresponding to a 524-fold increase (*p* = 0.0002). For Tob killing, the persister ratio of the parental strain was 10^−5.38±0.05^, whereas that of the *pth*-Tn mutant reached 10^−0.58±0.13^, corresponding to a 60,000-fold increase (*p* < 0.0001). For Cip killing, the persister ratio of the parental strain was 10^−3.17±0.14^, whereas that of the *pth*-Tn mutant reached 10^−1.00±0.12^, corresponding to a 144-fold increase (*p* < 0.0001). For Ofx killing, the persister ratio of the parental strain was 10^−3.84±0.03^, whereas that of the *pth*-Tn mutant reached 10^−1.43±0.18^, corresponding to a 258-fold increase (*p* < 0.0001). Collectively, these data demonstrate that the *pth*-Tn mutant displays broad-spectrum tolerance against a wide range of antibiotics.

Taken together, our data demonstrate that the *pth*-Tn mutant with a truncated C-terminal α-helix drastically attenuates strain growth and extends the lag phase time, which consequently results in substantially higher persister fractions upon exposure to multiple antibiotics.

### 3.3. Altered Profiles Consistent with a Dormant-like or Growth-Impaired Physiological State in pth-Tn Mutant Strain

**Figure 3:**
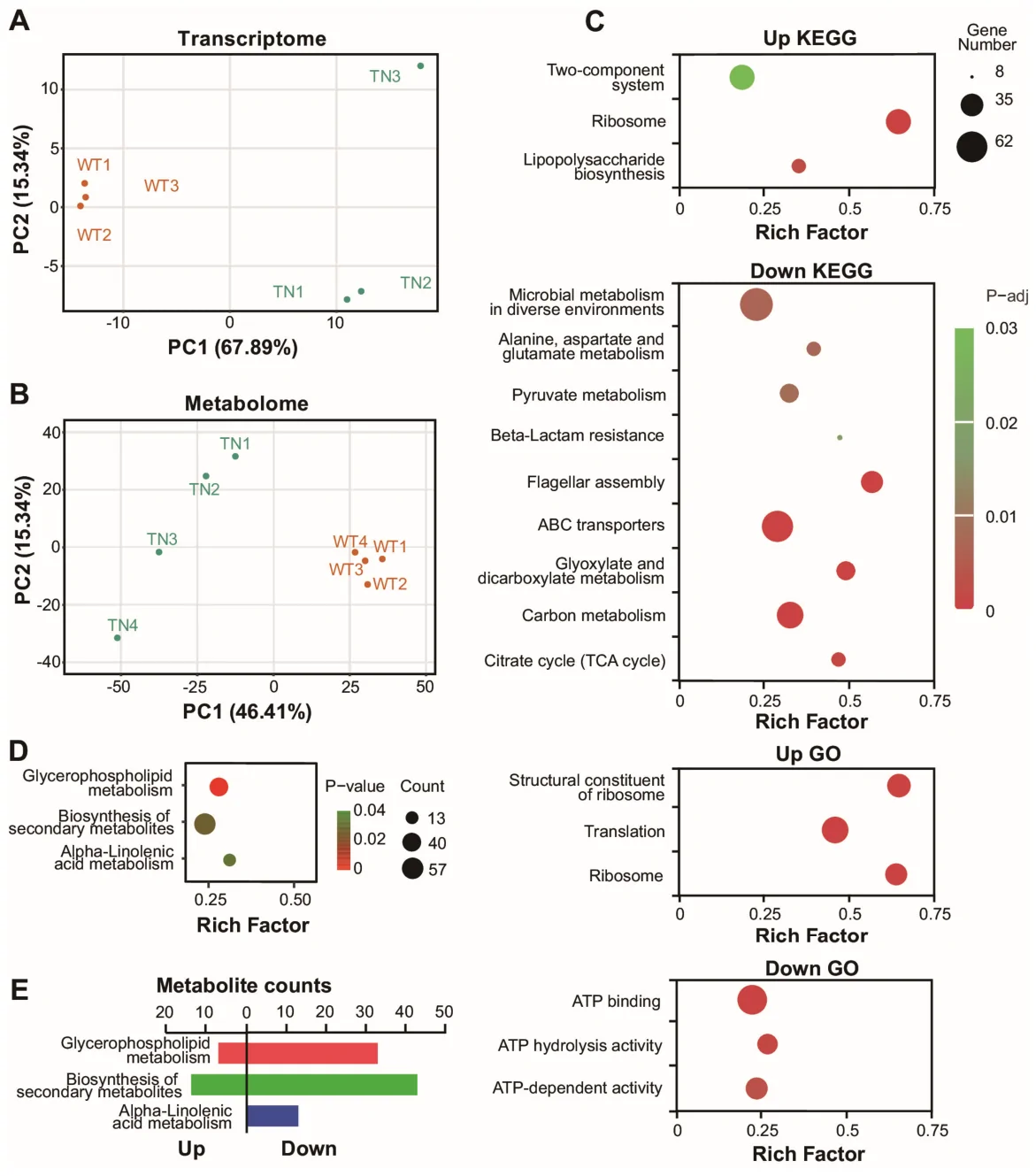
Transcriptomic and metabolomic analyses reveal changes consistent with a dormant-like state in the *pth*-Tn mutant strain. Principal component analysis (PCA) of gene expression profiles (**A**) and metabolite profiles (**B**) in wild-type parental strains and *pth*-Tn mutant strains. (**C**) Genes whose fold changes were greater than 2 and whose *P-adj* values were less than 0.05 in the *pth*-Tn mutant relative to the parental strain were selected for KEGG and GO enrichment analysis. (**D**) Metabolites whose fold changes were greater than 2, whose *p* values were less than 0.05 and whose VIP values were greater than 1 in the *pth*-Tn mutant relative to the parental strain were screened for KEGG enrichment analysis. (**E**) Counts of upregulated and downregulated differentially abundant metabolites for three KEGG pathways in Panel D. TN stands for *pth*-Tn. WT stands for MG1655 wild type.

Pth is a peptidyl-tRNA hydrolase, which maintains sufficient amounts of free tRNA and 50S ribosomes inside cells [21,22,23,24]. Therefore, the normal function of Pth is essential for bacterial growth. To further investigate the mechanism underlying the marked increase in persister cells upon *pth*-Tn mutation, we performed transcriptome sequencing and metabolomic analysis on the wild-type MG1655 strain and the *pth*-Tn mutant strain. Principal component analysis (PCA) of the transcriptomic and metabolomic data revealed a clear separation tendency between the parental strain and the *pth*-Tn mutant strain based on their global molecular profiles (Figure 3A,B).

There were 767 upregulated genes and 455 downregulated genes whose fold changes were greater than 2 and whose *P-adj* values were less than 0.05 in the *pth*-Tn mutant relative to the parental strain (Figure S2A,B). With respect to the upregulated genes, KEGG enrichment analysis revealed significant enrichment in pathways, including ribosome, two-component system and lipopolysaccharide biosynthesis (Figure 3C). GO enrichment analysis revealed significant enrichment in structural constituent of ribosome, translation and ribosome (Figure 3C). The *pth*-Tn mutant depletes the pool of functional ribosomes, which triggers robust compensatory upregulation of ribosome biogenesis in bacterial cells. Upregulation of the lipopolysaccharide biosynthesis pathway indicates decreased membrane permeability [34]. With respect to the downregulated differentially expressed genes, KEGG enrichment analysis revealed significant enrichment in pathways related to microbial metabolism in diverse environments, alanine/aspartate/glutamate metabolism, pyruvate metabolism, beta-lactam resistance, flagellar assembly, ABC transporters, glyoxylate and dicarboxylate metabolism, carbon metabolism and the citrate cycle (TCA cycle) (Figure 3C). GO enrichment analysis revealed significant enrichment in ATP binding, ATP hydrolysis activity and ATP-dependent activity (Figure 3C). Enrichment analysis revealed marked downregulation of energy metabolism in the *pth*-Tn strain. This represents a hallmark feature of growth arrest, which further accounts for the drastically elevated persister cell levels observed here.

There were 124 upregulated metabolites and 349 downregulated metabolites whose fold changes were greater than 2, whose *p* values were less than 0.05 and whose VIP values were greater than 1 in the *pth*-Tn mutant relative to the parental strain (Figure S2C,D). KEGG enrichment analysis was performed for all the differentially abundant metabolites, which revealed significant enrichment of three pathways: glycerophospholipid metabolism, biosynthesis of secondary metabolites, and alpha-linolenic acid metabolism (Figure 3D). The downregulated metabolites were enriched in all three pathways (Figure 3E). Global downregulation of the glycerophospholipid metabolism pathway severely impairs bacterial membrane biogenesis, leading to robust suppression of cell growth and the biosynthesis of biological macromolecules [35,36,37]. These findings are consistent with the reduced ATP activity revealed by GO enrichment analysis of downregulated genes in the transcriptome (Figure 3C). The downregulation of the biosynthesis of secondary metabolite pathways may be attributed to severe impairments of normal bacterial translational processes caused by the *pth*-tn mutation. To redirect energy resources toward the synthesis of abundant functional ribosomes, bacteria suppress secondary metabolic biosynthetic pathways. Repression of the alpha-linolenic acid metabolic pathway may reflect decreased membrane permeability [38], which is consistent with the concurrent downregulation of glycerophospholipid metabolism.

Taken together, the results of integrated transcriptome and metabolome profiling demonstrated molecular alterations consistent with a dormant-like or growth-impaired physiological state in the *pth*-Tn mutant.

### 3.4. Mutations in pth Are Correlated with Elevated Persister Fractions in Clinical Isolates

**Figure 4:**
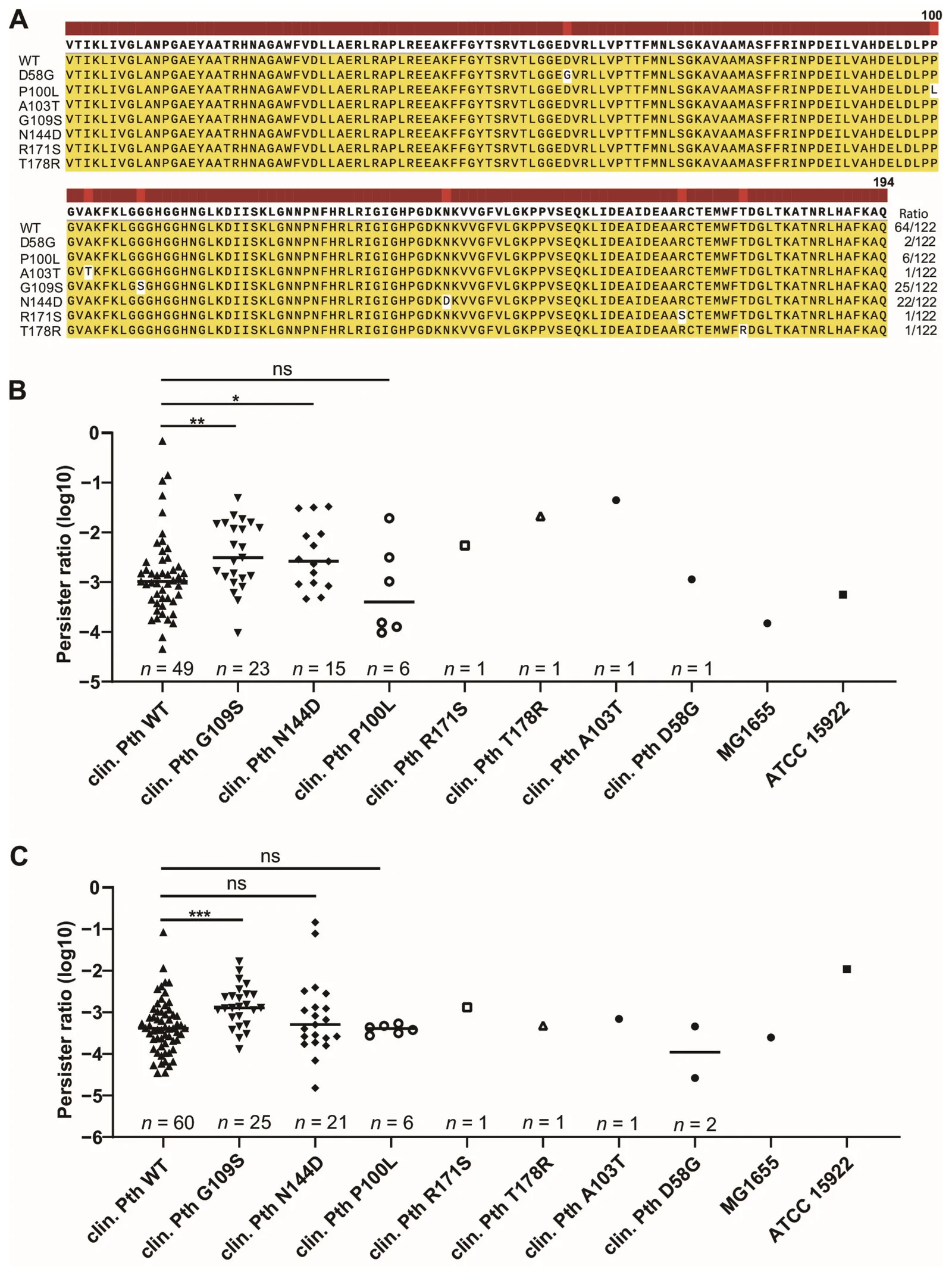
The *pth* G109S mutant exhibiting elevated persister fractions. (**A**) Protein sequence alignment of Pth point mutants and Pth-WT. (**B**,**C**) Clinical *Escherichia coli* isolates to be tested were inoculated into LB and incubated at 37 °C with shaking at 220 rpm for 12 h to stationary phase, followed by persister cell detection. Bacteria were treated with 150 μg/mL ceftazidime (Caz) for 3 h (**B**) or 2.5 μg/mL meropenem (MEM) for 3 h (**C**). Each data point represents the mean value of three biological replicates. The horizontal lines represent the median values. Significance was ascertained by the Mann-Whitney U test. A value of *p* < 0.05 was considered significant. “*” indicates significant differences (*, *p* < 0.05; **, *p* < 0.01; ***, *p* < 0.001). ns, not significant (*p* > 0.05).

We discovered that the essential gene *pth* tolerates minor mutations. Such mutations merely reduce the enzymatic activity or substrate affinity of the Pth protein rather than completely abolishing its function, thereby failing to induce bacterial lethality. Consistent with our observations, previous studies have isolated multiple *pth* mutant strains via artificial stress screening under laboratory conditions. Accordingly, we asked whether *pth* mutations occur in clinical *E. coli* isolates, and whether these mutations are correlated with the proportion of persister cells. Therefore, we isolated 122 clinical *E. coli* strains from clinical samples. Seven point mutants were identified through Sanger sequencing among these 122 isolates (Figure 4A). The isolation rates of strains with the wild type or each mutation type were as follows: WT, 64/122; D58G, 2/122; P100L, 6/122; A103T, 1/122; G109S, 25/122; N144D, 22/122; R171S, 1/122; and T178R, 1/122 (Figure 4A).

To determine the persister ratio of clinical *E. coli* isolates, we analyzed the minimum inhibitory concentrations (MICs) of multiple antibiotics. Caz (ceftazidime) or MEM (meropenem) was used to quantify the persister fractions (Table S2). For persister-fraction data obtained from Caz exposure (97 isolates with MIC < 8 μg/mL), compared with the wild-type (*n* = 49) *pth* clinical *E. coli* strains, the clinical *E. coli* strains carrying *pth* G109S (*n* = 23) and N144D (*n* = 15) point mutations presented markedly higher persister ratios (*p* = 0.013 and *p* = 0.045, respectively) (Figure 4B). However, clinical *E. coli* strains carrying *pth* P100L (*n* = 6) point mutations did not significantly differ from wild-type (*n* = 49) *pth* clinical strains (*p* = 0.375) (Figure 4B). For A103T, R171S and T178R, each mutation was identified in only one clinical isolate. We further performed point–biserial correlation analyses between *pth* mutations and persister fractions. The results revealed weak positive correlations between *pth* mutants and the persister fraction (*r_pb_* = 0.2931, *p* = 0.0125 for G109S; *r_pb_* = 0.2526, *p* = 0.044 for N144D). For persister-fraction data obtained from MEM exposure (117 isolates with MIC ≤ 0.25 μg/mL), compared with the wild-type *pth* (*n* = 60) clinical *E. coli* strains, the clinical *E. coli* strains carrying *pth* G109S point mutations (*n* = 25) presented markedly higher persister fractions (*p* = 0.0002) (Figure 4C). Clinical *E. coli* strains carrying *pth* N144D (*n* = 21) and P100L (*n* = 6) point mutations did not significantly differ from wild-type *pth* (*n* = 60) clinical strains (*p* = 0.450 and *p* = 0.888, respectively) (Figure 4C). The four mutations D58G, A103T, R171S and T178R were regarded as rare variants, and the limited number of corresponding isolates precluded statistical comparison. Point–biserial correlation analyses between *pth* G109S point mutations and persister fractions revealed positive correlations (*r_pb_* = 0.3936, *p* = 0.0002). Pairwise comparisons between these mutant groups and the wild-type group were exploratory. No multiple-comparison correction was applied, and the corresponding *p* values should be interpreted with caution.

To further quantify the independent contribution of G109S mutation to elevated Caz or MEM persister fractions, multiple linear regression models with stepwise confounder adjustment were constructed to eliminate confounding bias from antibiotic resistance phenotypes (Tobramycin, Minocycline, TMP-SMX, Moxifloxacin, Doxycycline) and sample source. In the Caz persister cohort (*n* = 85) (Table 1), G109S mutation significantly increased Caz persister fractions in the unadjusted Model 1 (β = 0.395, 95%CI 0.009–0.781, *p* = 0.045). As more confounders were added, the β value of G109S gradually decreased and the *p* value increased accordingly. In the full adjustment (Model 7), the association lost statistical significance (β = 0.312, 95%CI −0.085–0.709, *p* = 0.122). In the MEM persister cohort (*n* = 103) (Table 2), G109S remained significantly positively correlated with MEM persister fractions across all seven models. The effect size of G109S slightly rose after successive confounder adjustment, with the strongest independent effect in fully adjusted Model 7 (β = 0.567, 95%CI 0.246–0.888, *p* = 0.001). Collectively, within our clinical isolate collection, the G109S mutation displayed an independent association with higher persister fractions in a drug-specific manner. Confounders including antibiotic resistance and sample source markedly attenuated the statistical significance of G109S in the Caz cohort, whereas the association persisted and strengthened after full adjustment in the MEM cohort. These findings indicated that the *pth* G109S mutation is a factor affecting the persister fractions in clinical *E. coli* strains but that this effect is neither dominant nor sole one.

In summary, the persister fractions of clinical *E. coli* isolates carrying the G109S mutation of the *pth* gene were significantly greater than those of clinical isolates carrying the wild-type *pth* gene.

### 3.5. Phenotypic Analysis of Engineered pth Mutants and Complemented Clinical Isolates

**Figure 5:**
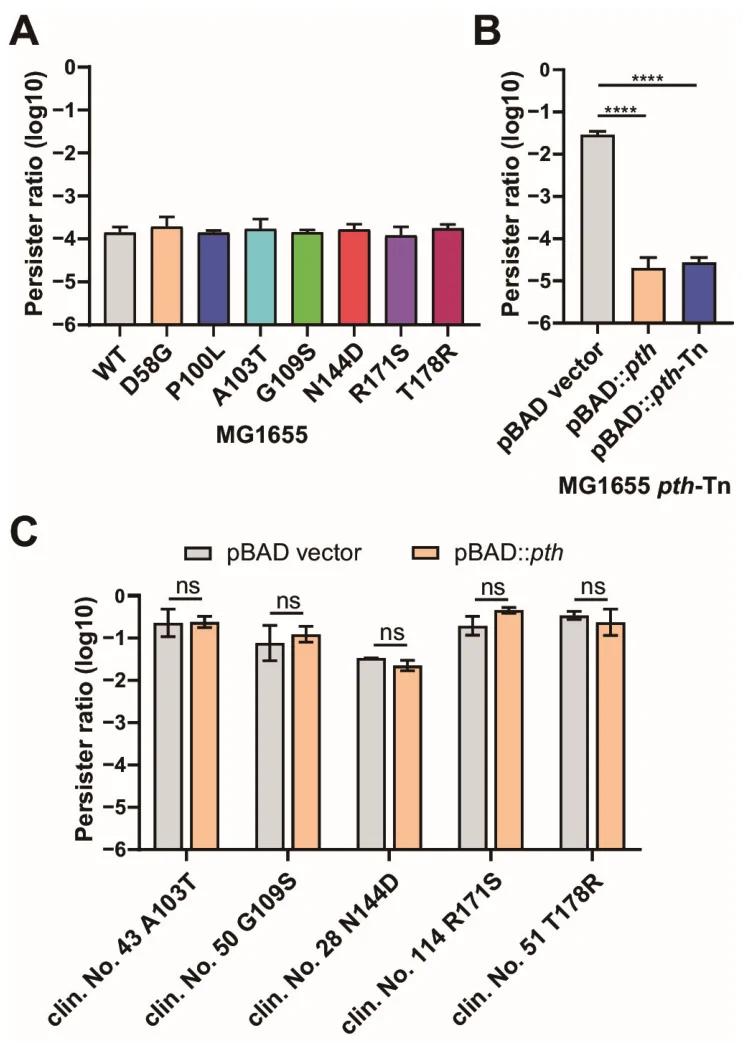
Persister fractions of mutant strains. (**A**) Persister cell assays of *pth* point mutants derived from MG1655. (**B**) Persister cell assays of *pth*-Tn mutants with *pth* complementation. (**C**) Persister cell assays of clinical strains with *pth* complementation. Overnight cultures were diluted 1:100 into fresh LB medium and incubated at 37 °C with shaking at 220 rpm until they reached the stationary phase, after which persister cells were detected. Significance was ascertained by two-tailed Student’s *t* test. The error bars represent the standard deviations of the mean from at least three biological replicates. A value of *p* < 0.05 was considered significant. “*” indicates significant differences (****, *p* < 0.0001). ns, not significant (*p* > 0.05).

To investigate how *pth* mutants affect persister formation, with particular focus on the G109S mutation, we constructed seven *pth* point mutant strains in the MG1655 strain based on the codon-optimized *pth* gene. Codon optimization of the *pth* gene did not affect persister levels (Figure S3A). Unfortunately, compared with those of wild-type MG1655, the persister fractions of these *pth* point mutant strains did not significantly increase (Figure 5A). qPCR was performed to determine the transcriptional levels of the pth gene in these mutant strains. The results revealed that the transcript levels of *pth* for all seven point-mutant strains were significantly elevated by approximately 10- to 20-fold (Figure S3B). In *pth* point-mutant strains, transcript levels of the mutated *pth* gene were markedly elevated. As shown in Figure 5B, overexpression of *pth*-Tn driven by its native promoter significantly reduced persister fractions in the *pth*-Tn strain. Thus, we hypothesize that even if these *pth* point mutations reduce Pth activity or substrate-binding capacity, abundant mutant Pth protein may compensate for this functional impairment.

We further employed overexpression of wild-type *pth* driven by its native promoter to investigate whether mutant *pth* represented the primary cause of elevated persister fractions in clinical isolates. Results showed that overexpression of wild-type *pth* reduced persister fractions of the MG1655 *pth*-Tn strain from 10^−1.54±0.08^ to 10^−4.69±0.24^ (*p* < 0.0001) (Figure 5C), validating the reliability of this experimental system. However, overexpression of wild-type *pth* in these clinical isolates failed to reduce persister fractions (Figure 5C). This may stem from the complex genetic backgrounds of clinical isolates, giving rise to diverse mechanisms for increased persister fractions. Taken together, these experimental results do not demonstrate that *pth* point mutations, including the G109S mutation, are sufficient to elevate persister fractions. Future studies could be designed to perform in vitro enzymatic activity assays that can eliminate the confounding effects of genetic background and variable gene expression levels.

## 4. Discussion

### 4.1. The pth-Tn Mutation Markedly Elevates Persister Cell Levels

It is widely recognized that persister cells contribute to a range of clinical problems, including antibiotic treatment failure and chronic and recurrent infections. Nevertheless, most studies on persister cells are limited to laboratory model strains [17,18,19,20]. Like most research groups, our team has carried out many persister-related experiments on model laboratory strains [12,13,39]. This study originates from persister enrichment screening based on an *E. coli* MG1655 transposon mutant library. Surprisingly, we performed three rounds of persister enrichment screening and identified a transposon insertion mutation in the essential gene *pth* (Figure 1A). Pth is a peptidyl-tRNA hydrolase and can hydrolyze ribosome-free peptidyl-tRNAs. Pth can also catalyze the release of premature peptidyl moieties from peptidyl-tRNA molecules and maintain sufficient free tRNA and 50S ribosomes inside cells [21,22,23,24]. Therefore, this protein is indispensable for normal bacterial growth [40,41]. Sequence analysis and structural modeling revealed disruption of the C-terminal α-helix of Pth in this mutant strain (Figure 1B,C). We hypothesize that disruption of the C-terminal α-helix of Pth retains normal catalytic activity but drastically weakens its substrate-binding capacity. This domain serves as the core structural element responsible for Pth recognition of the TΨC stem of tRNA [27]. For this reason, the *pth*-Tn mutant is not lethal and can survive. Similar results have been reported previously. In a laboratory evolution study, Anupama and Saeed performed repeated long-term screening using ampicillin combined with ciprofloxacin or kanamycin to evolve strains with higher persister levels. Two point mutations of *pth*, G101D and A170P, were identified in their work [28]. The G101D mutation was temperature sensitive and exhibited only 9.1% activity at 32 °C [23]. The function of the A170 residue of Pth is unknown.

We further characterized the *pth*-Tn mutant through growth curve measurement, lag time measurement and persister fraction quantification. Compared with the wild-type strain, this mutant showed limited growth and increased tolerance to various antibiotics, including β-lactam antibiotics (Amp, Cps and Caz), aminoglycoside antibiotics (Net and Tob) and fluoroquinolones (Cip and Ofx) (Figure 2). To explore the mechanism underlying the elevated persister levels in the *pth*-Tn mutant, we performed transcriptomic and metabolomic profiling. Transcriptome and metabolome analyses indicated enhanced ribosome biogenesis in the *pth*-Tn strain (Figure 3). Dysfunctional Pth fails to efficiently cleave and release peptidyl-tRNA from ribosomes. Thus, the abundance of functional ribosomes was decreased and could not support normal protein translation processes. Therefore, compensatory overproduction of ribosomes occurs to maintain translational capacity. This process consumes excessive amounts of cellular materials and energy. Consistently, transcriptomic and metabolomic data revealed multiple defects in the *pth*-Tn mutant, including in energy metabolism, membrane biogenesis, substrate transport and translational activity, among others. All these changes are consistent with characteristic features of a dormant-like or growth-impaired physiological state [28,42,43,44]. These observations collectively explain why the *pth*-Tn mutant exhibits higher persister fractions. We examined key global stress regulators in our transcriptome data. No significant expression changes were detected for *rpoS* and *crp* in the *pth*-Tn mutant, suggesting that the persister-related phenotype may be independent of these pathways.

Despite different experimental designs, our group and Saeed’s group converged on mutations in the same essential gene, *pth* [28]. This finding indicated that disruption of the essential *pth* gene readily drives increased persister formation. Moreover, it raises a possibility that if clinical strains circulating in human hosts harbor *pth* mutations during evolution, such mutants are likely linked to the persister phenotypes of clinical isolates.

### 4.2. The pth G109S Mutation Associated with Elevated Persister Fractions in Clinical E. coli Isolates

Heterogeneous subpopulations in bacterial populations are the basis of persister production [45]. The formation of such heterogeneous subpopulations is governed by genes with heterogeneous expression patterns. In laboratory-based fundamental research, these heterogeneously expressed genes that drive persister formation are generally regarded as molecular signatures of persister subpopulations [16,46]. However, such heterogeneity cannot be translated into simple, low-cost, high-throughput clinical applications and remains confined to basic laboratory research. For instance, cells with elevated intracellular (p)ppGpp levels are predisposed to persister formation [47,48,49]. Nevertheless, clinical laboratories lack the ability to perform single-cell quantification of (p)ppGpp in clinical isolates.

The quantification of persister fractions in clinically isolated strains is highly important, as it can provide critical references for clinicians to formulate medication regimens. However, most current laboratory investigations into the mechanisms underlying persister formation have limited utility for the clinical screening of strains with higher persister fractions. This is because of the intricate evolutionary trajectories of clinical isolates, which accumulate more mutations than model strains do. These genetic variations may have diverse impacts on persister formation. Accordingly, persister mechanisms uncovered in model strains cannot faithfully recapitulate the phenotypes seen in clinical isolates. Because clinical isolates, such as the hipA7 mutant [50], accumulate extensive mutations, we believe that investigating the correlations between these mutations and persister formation and developing mutation-targeted assays for persister quantification or risk prediction could be a research strategy with clinical translational value.

Based on our previous *pth* screening work together with research from the Saeed group, we hypothesized that *pth* mutations in clinical isolates would be associated with elevated persister fractions. Therefore, we collected 122 clinical *E. coli* strains and identified seven distinct *pth* point mutations. One important limitation of this study is the absence of WGS- or MLST-based phylogenetic analysis. We were therefore unable to evaluate bacterial population structure and lineage-related confounding factors. Such underlying genetic relatedness among isolates could potentially distort the observed statistical association between the *pth* mutations and persister fractions. The clinical strain collection was derived from multiple hospital wards, different patients, and different clinical specimen types (Table S2). This epidemiological diversity may lower the risk of widespread clonal spread, yet it cannot definitively exclude genetically related lineages.

Further analyses revealed that compared with strains with wild-type *pth*, clinical isolates carrying *pth* G109S mutation presented significantly higher persister fractions. Positive correlations were observed between the *pth* G109S mutation and persister fractions (Figure 4). To exclude the influences of confounding factors, we incorporated sample source and five antibiotics belonging to distinct classes, including tobramycin (aminoglycoside), minocycline and doxycycline (tetracyclines), moxifloxacin (fluoroquinolone), and TMP-SMX, into multiple linear regression models. The G109S mutation was independently associated with elevated persister levels (Table 1 and Table 2). Unfortunately, the number of strains carrying D58G, A103T, R171S and T178R mutations was too small to determine whether these mutations are associated with elevated persister fractions in clinical isolates. Further investigations with more clinical strains are needed to validate this possibility in future studies. Further detailed investigations are still required before the *pth* G109S mutant can be regarded as a potential biomarker for high-persister phenotypes. These investigations should include evaluation of predictive performance, independent validation, assessment of sensitivity, specificity, positive predictive value, negative predictive value, and ROC curve analysis, among other approaches. Such gene point mutations in clinical isolates can be screened in a rapid, low-cost and high-throughput approach using molecular biological techniques, which demonstrates great translational prospects. Nevertheless, relying solely on the *pth* G109S mutant cannot accurately predict the persister levels of clinical strains. Future research should identify more relevant genetic variants and implement multivariate comprehensive analyses. We should focus particularly on evolutionary mutations within essential genes.

To explain why clinical isolates carrying *pth* G109S mutant exhibit higher persister levels than wild-type *pth* clinical strains, we constructed corresponding point-mutant strains in the MG1655 background. Given that *pth* is an essential gene, codon-optimized *pth* fragments were used as templates to generate site-directed *pth* mutations via the λ-Red recombination system. However, our results revealed that compared with those in wild-type MG1655, the persister fractions in all these *pth* mutants in MG1655 did not significantly differ. This may stem from markedly elevated transcript levels of these mutant *pth*. On the other hand, complementation with wild-type *pth* failed to reduce persister fractions in clinical isolates harboring *pth* point mutations. These findings fail to establish that the G109S mutation can directly cause elevated persister fractions. Future in vitro biochemical assays could be performed to verify whether these mutants impair Pth enzymatic activity or substrate binding. Notably, previous in vitro investigations have not identified any functional linkage between these seven residues and the catalytic activity or substrate affinity of Pth [23,25,26,27].

## 5. Conclusions

Transposon insertion mutant of the essential gene *pth* exhibited significantly elevated persister levels, accompanied by features consistent with a dormant-like or growth-impaired physiological state.

The *pth* G109S mutation is associated with enhanced persister phenotypes in clinical *E. coli* strains.

## Figures and Tables

**Table 1:** Multiple linear regression for associations between *pth* G109S mutant and Caz-derived persister fractions.

	Model 1	Model 2	Model 3	Model 4	Model 5	Model 6	Model 7
Adjusted R^2^	0.043	0.074	0.065	0.087	0.076	0.088	0.079
n	85	85	85	85	85	85	85
β	0.395	0.383	0.381	0.370	0.360	0.331	0.312
*p*	**0.045**	**0.048**	0.051	0.055	0.068	0.092	0.122
95% CI	(0.009, 0.781)	(0.003, 0.763)	(−0.001, 0.763)	(−0.007, 0.748)	(−0.027, 0.746)	(−0.056, 0.718)	(−0.085, 0.709)

Model 1 included G109S and N144D as predictor variables. Model 2 added tobramycin susceptibility. Model 3 further included minocycline susceptibility. Model 4 further included TMP-SMX susceptibility. Model 5 further included sample source. Model 6 further included Moxifloxacin susceptibility. Model 7 further included Doxycycline susceptibility. Wild-type *pth* and major variants (G109S and N144D) were included in the multiple linear regression analysis. Samples with missing values were excluded, yielding a final dataset of *n* = 85.

**Table 2:** Multiple linear regression for associations between *pth* G109S mutant and MEM-derived persister fractions.

	Model 1	Model 2	Model 3	Model 4	Model 5	Model 6	Model 7
Adjusted R^2^	0.072	0.064	0.059	0.050	0.048	0.045	0.057
n	103	103	103	103	103	103	103
β	0.490	0.491	0.498	0.498	0.522	0.526	0.567
*p*	**0.002**	**0.002**	**0.002**	**0.002**	**0.002**	**0.001**	**0.001**
95% CI	(0.182, 0.798)	(0.181, 0.801)	(0.187, 0.809)	(0.185, 0.810)	(0.205, 0.840)	(0.208, 0.845)	(0.246, 0.888)

Model 1 included G109S and N144D as predictor variables. Model 2 added tobramycin susceptibility. Model 3 further included minocycline susceptibility. Model 4 further included TMP-SMX susceptibility. Model 5 further included sample source. Model 6 further included Moxifloxacin susceptibility. Model 7 further included Doxycycline susceptibility. Wild-type *pth* and major variants (G109S and N144D) were included in the multiple linear regression analysis. Samples with missing values were excluded, yielding a final dataset of *n* = 103.

## Data Availability

The intermediate files and raw bulk RNA-seq data generated in this study have been deposited in the NCBI Gene Expression Omnibus (GEO) under accession number GSE337979, https://www.ncbi.nlm.nih.gov/geo/query/acc.cgi?acc=GSE337979 (accessed on 12 August 2026). The intermediate files and raw metabolomics data generated in this study have been deposited in the Metabolomics Workbench (datatrack_id:7814, study_id:ST005025), https://dev.metabolomicsworkbench.org:22222/data/DRCCMetadata.php?Mode=Study&StudyID=ST005025&Access=VaqM9397 (accessed on 18 August 2026). The analytical scripts used in this study have been uploaded to GitHub, https://github.com/123456yxd/TN-seq (accessed on 18 August 2026).

## Supplementary Materials

The following supporting information can be downloaded at: https://www.mdpi.com/article/10.3390/pathogens15080875/s1.
