# Supplementary material for: Association of pth Variants with Elevated Persister Fractions in Clinical Escherichia coli Isolates
Source: Pathogens. 2026 Aug 20;15(8):875. doi: 10.3390/pathogens15080875 (PMC13516025; doi:10.3390/pathogens15080875)
Supplement: Supplementary file 1 [file pathogens-15-00875-s001.zip › Supplementary Text (Text S1 and S2).pdf]

Text S1.

pth-Tn sequence in the Tn library (*pth*) strain

GTGACGATTAAATTGATTGTCGGCCTGGCGAACCCCGGTGCTGAATACGCCGCAACGC
GACATAATGCTGGTGCCTGGTTCGTTGACTTACTGGCAGAGCGTTTGCGCGCTCCGCTG
CGCGAAGAGGCTAAATTCTTTGGTTATACTTCGCGAGTCACTCTTGGAGGCGAAGATGT
CCGCCTGTTAGTCCCGACTACATTTATGAATCTCAGCGGCAAAGCCGTTGCGGCGATGG
CCAGTTTTTTCCGCATTAATCCGGACGAAATTCTGGTGGCCCACGACGAACTGGATCTG
CCTCCTGGCGTCGCCAAATTTAAATTGGGCGGTGGCCATGGTGGTCACAATGGACTGA
AAGACATCATCAGTAAATTGGGTAATAACCCTAACTTTCACCGTTTACGCATCGGAATC
GGTCATCCGGGCGATAAAAATAAAGTTGTCGGTTTTGTGTTAGGCAAACCGCCTGTTAG
TGAACAGAAGTTAATTGATGAAGCCATTGACGAAGCGGCGCGTTGTAACAGGTTGGAT
GATAAGTCCCCGGTCTCTAGAATTCACCATAGACTACAAAGACCATGA

Text S2.

Codon-optimized sequence of the *pth* gene

GTGACGATTAAATTGATTGTCGGCCTGGCGAACCCCGGTGCTGAATACGCCGCAACGC
GACATAATGCTGGTGCCTGGTTCGTTGACTTACTGGCAGAGCGTTTGCGCGCTCCGCTG
CGCGAAGAGGCTAAATTCTTTGGTTATACTAGTAGGGTtACGTTAGGGGGtGAGGACGT
TCGTTTACTGGTTCCaACcACtTTcATGAACTTAtctGGtAAgGCgGTgGCcGCcATGGCgtctTT
cTTtCGtATcAAcCCaGAtGAgATcttaGTtGCcCAtGAtGAgttaGActtaCCaCCaGGtGTgGCgAAgT
TcAAgcttGGtGGcGGtCAcGGcGGcCAtAAcGGtttaAAgGAtATtATttccAAgcttGGcAAcAAtCCa
AAtTTcCAtCGcctgCGtATtGGtATtGGcCAcCCaGGtGAcAAgAAcAAgGTgGTgGGcTTcGTtctg
GGtAAgCCaCCgGTgtccGAgCAaAAactgATcGAcGAgGCgATcGAtGAgGCcGCcCGcTGcACc
GAgATGTGGTTcACcGAcGGtcttACgAAgGCgACcAAtCGtcttCAtGCgTTcAAgGCcCAgTAA
