# Supplementary material for: Association of pth Variants with Elevated Persister Fractions in Clinical Escherichia coli Isolates
Source: Pathogens. 2026 Aug 20;15(8):875. doi: 10.3390/pathogens15080875 (PMC13516025; doi:10.3390/pathogens15080875)
Supplement: Supplementary file 1 [file pathogens-15-00875-s001.zip › Supplementary Figure legends.pdf]

Figure S1. Time-kill curves of multiple *E. coli* isolates exposed to Caz. Panels A-D represent time-kill curves for strains MG1655, ATCC 25922, Clin. No. 50 and Clin. No. 28, respectively. Overnight cultures were diluted 1:100 into fresh LB medium and incubated at 37 °C with shaking at 220 rpm until they reached the stationary phase. The bacterial suspension was diluted 20 fold into fresh LB medium supplemented with 150 μg/mL Caz. At defined time points, aliquots of the culture were collected, washed with PBS, and processed for CFU counting. The error bars represent the standard deviations of the mean from at least three independent experiments.

Figure S2. Volcano plots and heatmaps derived from transcriptomic and metabolomic data of the *pth*-Tn mutant and WT strain. (A) Volcano plots derived from transcriptomic data. Genes with $|\log_2 FC| \geq 1$ and *P-adj* < 0.05 were defined as differentially expressed genes. (B) Volcano plots derived from the metabolomic data. Metabolites with $|\log_2 FC| \geq 1$, *P* value < 0.05 and VIP ≥ 1 were defined as differentially accumulated metabolites. (C) Heatmap of all differentially expressed genes identified by transcriptome sequencing. (D) Heatmap of all differentially accumulated metabolites from the metabolomic analysis. Raw relative abundances of differential metabolites were processed by unit variance (UV) scaling. TN stands for *pth*-Tn. WT stands for MG1655.

Figure S3. Phenotypic characterization of *pth* mutant strains derived from MG1655. (A) Persister cell assays of *pth*-WT and *pth*-opt. derived from MG1655. Overnight cultures were diluted 1:100 into fresh LB medium and incubated at 37 °C with shaking at 220 rpm until they reached the stationary phase, after which persister cells were detected. (B) Relative mRNA levels of the *pth* gene. Overnight cultures were diluted 1:100 into fresh LB medium and incubated at 37 °C with shaking at 220 rpm until they reached the stationary phase, after which total RNA was extracted and reverse-transcribed into cDNA for subsequent qPCR analysis. Significance was ascertained by two-tailed Student's *t* test. The error bars represent the standard deviations of the mean from at least three independent experiments. A value of *P* < 0.05 was considered significant. "*" indicates significant differences (**, *P* < 0.01; ***, *P* < 0.001; ****, *P* < 0.0001). ns, not significant (*P* > 0.05).
